# Selectivity in social and asocial learning: investigating the prevalence, effect and development of young children's learning preferences

**DOI:** 10.1098/rstb.2015.0189

**Published:** 2016-03-19

**Authors:** Emma Flynn, Cameron Turner, Luc-Alain Giraldeau

**Affiliations:** 1 Centre for the Coevolution of Biology and Culture, School of Education, Durham University, Durham, DH1 1TA, UK; 2 Département des Sciences Biologiques, L'Université du Québec à Montréal, Montréal, Quebec, Canada, H2X 1Y4

**Keywords:** innovation, modification, learning preference, asocial learning, social learning

## Abstract

Culture evolution requires both modification and faithful replication of behaviour, thus it is essential to understand how individuals choose between social and asocial learning. In a quasi-experimental design, 3- and 5-year-olds (176), and adults (52) were presented individually with two novel artificial fruits, and told of the apparatus' relative difficulty (easy versus hard). Participants were asked if they wanted to attempt the task themselves or watch an experimenter attempt it first; and then had their preference either met or violated. A significant proportion of children and adults (74%) chose to learn socially. For children, this request was efficient, as observing a demonstration made them significantly quicker at the task than learning asocially. However, for 5-year-olds, children who selected asocial learning were also found to be highly efficient at the task, showing that by 5 years children are selective in choosing a learning strategy that is effective for them. Adults further evidenced this trend, and also showed selectivity based on task difficulty. This is the first study to examine the rates, performance outcomes and developmental trajectory of preferences in asocial and social learning, ultimately informing our understanding of innovation.

## 1. Introduction

Cultural evolution requires both accurate replication (high-fidelity transmission) of cultural behaviours and products, as well as innovation, which improves those behaviours and products [1]. Much research has focused on young children's sophisticated high-fidelity transmission via imitation, demonstrating that children have far more capacity for engaging in the behaviour necessary for cultural acquisition and transmission than other closely related species [2]. However, little research has directly examined how more asocial processes influence learning in children, and specifically, what capacities are present to allow change to cultural practices.

Innovation has many forms [3–5]. For example, in independent invention individuals discover a solution to a problem, such as creating a hook from a pipe-cleaner to extract a bucket from an otherwise inaccessible location [6]. By contrast, in modification, individuals build on the behaviour of others to create something new, which is more effective or efficient, a process critical for cultural evolution outlined above [2]. Each form of innovation draws differentially on social and asocial learning: modification requires the social learning of others' current behaviour, which is then changed asocially to create a novel behaviour. Similarly, all independent inventors bring to a task, undertaken asocially, a wealth of experience much of which is acquired socially. It has been argued that only once such novel behaviours are copied by others can they be considered to be an innovation [7]. Yet critically, irrespective of the definition used, for an individual to innovate s/he must start by do something ‘new’ and not simply copy with high fidelity the behaviour of others.

An attraction to social information in humans is present from birth [8]. Yet, children are selective regarding the context in which they reproduce socially learned information and which part of this information they reproduce. For instance, children are more likely to adopt behaviour when the observed behaviour is effective [9,10], and when a task is difficult and less familiar [11–14]. The identity of the model is also an influencing factor in whether one copies with high fidelity or produces something different [15]. Whiten & Flynn [16] investigated whether children copy the behaviour of others or innovate in a naturalistic setting of a playgroup [16]. They presented children with a novel puzzle box containing a reward, and found the overwhelming mechanism for learning was social, mostly through observation (64%), and occasionally teaching (5%) or a combination of the two (14%); independent learning was rare in comparison (17%). Similarly, in a more controlled setting where children were presented with a demonstration by a peer, in a diffusion chain design, Flynn & Whiten [17] found that only 1 out of 80 children attempted a behaviour that was not in line with what they had witnessed [17].

Our worlds are filled with social information, such that when children wandered freely around their playgroup in Whiten & Flynn [16], they witnessed their peers retrieving the reward from the novel apparatus that was presented to the group, and social learning took place [16]. What is unclear is whether children would have chosen to learn socially had they been given the choice of learning for themselves (asocially) or learning from others (socially). Although learning from others does not remove the possibility of innovation, as evident in modification, studying the preference to learn asocially as opposed to socially has important implications for our understanding of cultural learning. Only when individuals depart from high fidelity copying can innovations be produced. Therefore, the question of when the choice to learn socially versus asocially occurs in development and how this affects subsequent behaviour informs us about the development of skills and preferences necessary for innovation.

Although much research has focused on the ubiquity of social learning in children, we know of noone who has focused on the occurrence of asocial learning preferences and a comparison of the effects of this choice on performance. It has been shown that independent innovation is a relatively late-developing capacity [18–20] and modification is a rare response for children [16]. Factors such as functional fixedness [21], explicit instruction [22], prior social information [14] and task structure [23] all add to this rarity. Perhaps learning style preference can be added to this list.

Children may be tactical in their choice of social versus asocial learning. A decision to adopt one of these strategies over the other may be mediated by the difficulty of a task: for more difficult tasks children may decide to learn from others, thus reducing the likelihood of trial and error learning, whereas, for an easy task children may decide to bypass social information. Here we assessed the role of task difficulty in learning preference by presenting participants with a task labelled as ‘easy’ or ‘hard’. Recent work by Mesoudi [24] with adults lies in contrast to the predictions above. In a complex artefact-design task, approximately three-quarters of adults chose to learn asocially rather than socially, despite the complexity of the task and the fact there was a financial reward for success. These adults' preferences for asocial learning are a stark contrast with the rate of social learning described above for young children; this difference may be because in Mesoudi's experiments adults were in the position to choose which learning style they drew upon, or may be developmental. This study includes two child samples and an adult sample to investigate whether and, if so, how preferences for asocial or social learning change over the lifespan. Additionally, including adults in our study allowed us to ask about perceptions of task difficulty and assess more directly how these perceptions affected learning style preference and performance. We also used artificial fruit (AF) tasks, and so can consider if differences in learning preferences are witnessed when one has direct, as opposed to virtual, access to an artefact and models.

A further, critical question regarding individuals' preferences for either social or asocial learning is whether they provide efficient solutions to the task. Furthermore, to what degree do individuals at different ages recognize if social versus asocial learning is needed to be successful? Generally, social learning is extremely efficient not only in situations that are life-threatening, such as trying new untested foods, but also in more mundane novel tasks. For example, in studies of imitation, children who witness a demonstration show significantly higher rates of success when attempting the task, than individuals who attempt the task asocially [9,11,16]. Also while independent invention rates are low in children, when presented with a full or partial demonstration, children of the same age show high levels of success [18,20]. What is not clear is whether children who choose to learn asocially do so because they benefit from learning in this way compared with individuals who learn this way but would rather learn socially. To investigate the role of learning style preference on performance the sample were divided, half receiving their learning style preference and half receiving the alternative learning style; for example, requesting asocial learning but receiving social learning. By comparing the performance latencies across these groups we could establish if a learning style preference informs one's performance. For example, are those who chose asocial learning and receive asocial learning faster than those who chose social learning but learned asocially?

## 2. Material and methods

### (a) Participants

Seventy-eight 3-year-olds (39 female, *M* = 44.65 months, s.d. = 3.48 months) and 98 5-year-olds (44 girls, *M* = 66.74, s.d. = 3.37) from nurseries and schools in the northeast of England participated. For an adult comparison, 52 undergraduate students from a university in the northeast of England (29 female; *M* = 20.58 years, s.d. = 1.99 years) also participated (reimbursed with course credits or £5). Informed consent was acquired from all participants.

### (b) Design

**Figure 1.**
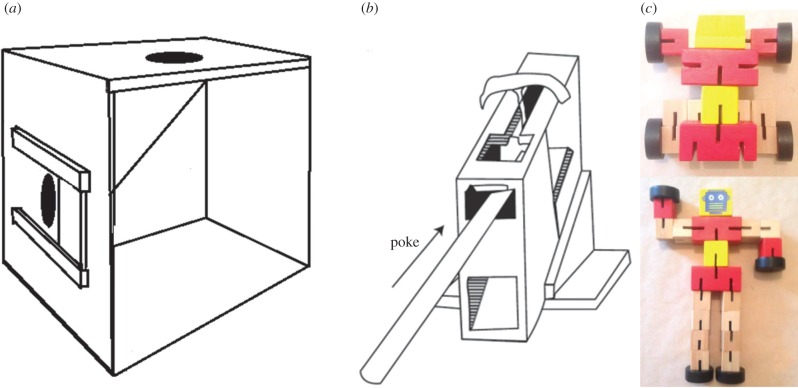
The tasks used: (*a*) ‘easy’ slide-door box, (*b*) ‘hard’ panpipes and (*c*) transformer task.

A 4 (preferred learning strategy congruency) × 2 (AF difficulty) mixed factorial design was employed. Preferred learning strategy congruency was made up of two features, children's learning preference (social versus asocial) and preference congruency (preference met versus preference violated), which resulted in four between-group conditions: (i) chose-social–received-social, (ii) chose-social–received-asocial, (iii) chose-asocial–received-asocial and (iv) chose-asocial–received-social. AF difficulty was manipulated within-participants over two levels: easy versus difficult. Adults received the same procedure; however, at the end of testing adults also completed a further task, a complex wooden block puzzle (Transformer task, figure 1).

### (c) Apparatus

Two AF tasks, the slide-door box (SB, figure 1*a*; [25]) and panpipes (PP, figure 1*b*; [26]) were used. For both tasks a series of actions needed to be performed to remove defences that held a reward inside (figure 1). The ‘difficulty’ distinction was supported by previous research with children of a similar age to those in this study who worked on the SB and PP asocially (previous success rate on the ‘easy’ SB = 75% and the ‘hard’ PP = 19%) [27]. Both AFs were adapted so that only one of the possible extraction methods was available: the door in the SB could only be pushed left-to-right and the poke method for the PP could be used. In addition, adults were also presented with a wooden transformer toy (Fidgetz Transformable Warriors, figure 1*c*), comprising a wooden block puzzle that can be transformed from a car-shape to an anthropomorphic form. Rather than stating the difficulty of the task, we asked participants to assess task difficulty: adults responded to the item ‘How difficult do you think this [Transformer] task will be?’ on a 7-point Likert scale anchored at 1 (easy) to 7 (difficult), *M* = 3.96, s.d. = 1.34.

### (d) Procedure

Testing took place individually in a quiet room within a participant's nursery, school or university. Initially, a participant was told, ‘I have two puzzle boxes. Each one has a sticker inside, one is easy to use, one is hard. Let's play with the easy/hard one first’. Upon seeing the AF the participant was asked, ‘Do you want to have a go, or do you want to watch me do it first?’ The clauses of 'having a go first' and 'watching first' as well as the order of presentation of the two AFs were counterbalanced across the sample. Once the participant made her/his request, s/he was randomly assigned to one of two conditions: having the request met by receiving either the learning style selected (social or asocial), or violated by being allocated to the alternative learning style (resulting in the four conditions outlined in the design). For the chose-asocial–received-asocial condition the experimenter said, ‘Okay, you have a go first; here you go’, and in the chose-social–received-social, ‘Okay, I'll have a go first; you can watch me’. In the chose-social–received-asocial the experimenter said, ‘Hmmm, actually, why don't you have a go first; here you go’. In the chose-asocial–received-social condition he said, ‘Hmmm, actually, why don't you watch me have a go; alright, watch’. After the demonstrations the experimenter said, ‘Okay, now you can have a go.’ Prompts of ‘keep watching’ or ‘have a go’ were used, as appropriate, to encourage participants if they became distracted. Children showed no signs of concern about the violation of request, and happily followed the experimenter's instructions. Time taken to complete the task (latency from first touch to success or 6 min cut-off) was measured. For adult participants, when completing the Transformer task, they were shown an image of the transformation (anthropoid to car) they had to perform, and were told: ‘Your task is to transform this transformer from its current state into the state shown on the box’.

## 3. Results

Initially, we address whether our distinction of task difficulty was upheld with this sample. Second, we assess whether individuals preferred to learn socially or asocially, and whether this was related to age, task difficulty and order of presentation. Finally, we address whether learning style preference with regard to learning style received affected performance in terms of latency to solution and rate of success. The benefit of our design was to measure both learning style preference and how this affected subsequent performance; however, this also meant we had a quasi-experimental design in which learning preference subdivided participants into conditions for the performance measures. For children, after an initial data collection (*n* = 96), preliminary analyses were conducted and it was found that a substantial bias for social learning preference meant conditions needed to be filled-up further to allow proper tests of performance. Therefore, a second round of data collection was undertaken (*n* = 138, preference-only *n* = 38; further descriptive statistics of conditions can be found in the electronic supplementary material, S1).

### (a) How difficult were these tasks for our participants?

Baseline performances of children on each task are from the first trial on the asocial learning conditions, as this is most comparable data to those that we used to measure task difficulty before the study. For the easy SB, 83% of 3-year-olds and 100% of 5-year-olds were successful, taking a mean of 89 s (s.d. = 137 s) and 10 s (s.d. = 14 s), respectively. For the hard PP, 75% of 3-year-olds and 89% of 5-year-olds were successful, taking a mean of 157 s (s.d. = 156 s) and 86 s (s.d. = 134 s), respectively. The difference in performance in the current study compared with Hopper *et al.* [27] was unexpected as the children were a similar age and from a similar cohort. The difference in performance across these two studies could have been because the panpipes in the current study were slightly modified (one of the two-action mechanisms, the T-bar, was removed as this was not required). Removing the T-bar may make it easier to identify the alternative method (pushing the obstructing block out of the way). There were no significant differences in latency according to AF difficulty in the asocial learning conditions for children, *t*_40_ = 1.76, *p* = 0.086, respectively. For the SB task, adults took a mean of 1 s (s.d. = 0.67 s), with a maximum time of 3 s. For the PP task, adults took a mean of 14 s (s.d. = 17 s), with a maximum time of 52 s, taking significantly longer, *t*_15_ = 2.33, *p* = 0.034. The Transformer task took a mean of 6 s (s.d. = 2 s), with a maximum time of 12 s.

### (b) Did the participants prefer to learn socially or asocially?

For the first trial, both 3- and 5-year-olds showed a significant preference to learn socially rather than asocially (3-year-olds, 69% social preference, *t*_77_ = 3.28, *p* = 0.001; 5-year-olds, 82% social preference, *t*_97_ = 6.16, *p* < 0.001). Fisher's exact test showed that the difference in learning preference between the age groups was not significant, *p* = 0.075. For 3-year-olds, AF difficulty had no effect on learning preference; 70% asked to learn socially with the easy SB, and 68% with the hard PP, Fisher's exact test, *p* = 1.000. Similarly, for 5-year-olds, 79% asked to learn socially with the easy SB, and 84% with the hard PP, Fisher's exact test, *p* = 0.600. Adults also showed a preference to learn socially (67% social preference, *t*_51_ = 2.36, *p* = 0.018) in the first trial. There was no significant effect of AF difficulty on learning style preference; 62% requested social learning for the easy SB and 73% for the hard PP, Fisher's exact test, *p* = 0.555.

In the children's second trial there was an analogous preference to learn socially, 68% of 3-year-olds, *t*_71_ = 4.24, *p* = 0.007, and 71% for 5-year-olds, *t*_65_ = 4.06, *p* = 0.001 selecting social learning, with no difference in preference between ages, Fisher's exact test, *p* = 0.586. Three-year-olds showed no differential preference for learning socially on either the easy SB, 64% or hard PP, 65%, Fisher's exact test, *p* = 0.803; nor did 5-year-olds, 61% and 82%, respectively, Fisher's exact test, *p* = 0.102.

For adults in their second trial, there was no significant difference in preference: 50% requested social learning, *t*_51_ = 0.00, *p* = 1.000. There was a marginally significant preference to learn socially when receiving the hard PP, 65%, compared with the easy SB, 35%, Fisher's exact test, *p* = 0.051. When presented with the Transformer task there was a significant preference to learn socially, 65%, *t*_51_ = 2.08, *p* = 0.038. There was a significant difference in preference choice based on task assessment, with those selecting social information assessing the task as more difficult (*M* = 5.00, s.d. = 0.97) than those selecting asocial (*M* = 3.41, s.d. = 1.18), *t*_50_ = 4.88, *p* < 0.001.

### (c) Did learning style preference and learning style method affect performance?

#### (i) Children's task latency

Using a three-way analysis of variance (ANOVA; learning preference: social or asocial; congruency: preference met or violated; age: 3 or 5 years), we examined the effect of children's learning style choice on their task performance (latency) in the first trial. AF difficulty was entered as a non-interacting factor, as it did not contribute to explaining additional variance by interaction (as seen in table 1; electronic supplementary material, S2). To account for any influence of uneven cell sizes and variance, the same analyses were run with procedures robust to the violation of this assumption (for test of assumption, along with bootstrapping, 1000 resamples, and Games–Howell *post hoc* analyses, log-transformed versions of analyses and equivalent non-parametric test were performed, see electronic supplementary material, S3), as the same pattern of results was produced. For ease of interpretation, the original analyses are reported here.

**Table 1.** Analysis of variance of the children's task latency (*N* = 138).

	d.f.	*F*	*p-*value
model^a^	8	11.20	0.001**
age group	1	6.46	0.012*
AF difficulty	1	6.17	0.014*
preference congruence	1	9.91	0.002*
learning preference	1	2.94	0.089
age group × learning preference	1	1.33	0.251
age group × preference congruence	1	0.02	0.896
learning preference × preference congruence	1	39.21	0.001**
age group × learning preference × preference congruence	1	4.22	0.042*

^a^*R*^2^ = 0.41.

**p < 0.001; *p < 0.05.

Unsurprisingly, 5-year-olds were significantly faster at the task than 3-year-olds and the easy SB was completed faster than the hard PP. Children who had their learning style preference met (*M* = 85 s, s.d. = 126 s) were significantly faster than those receiving their non-preferred learning style (*M* = 187 s, s.d. = 160 s). Stated learning style preference, whether social (*M* = 153 s, s.d. = 156 s) or asocial (*M* = 91 s, s.d. = 133 s), produced no significant effect. However, both learning preference and preference congruence interacted significantly, thus there was a significant three-way interaction between age group, learning preference and preference congruence.

**Figure 2.**
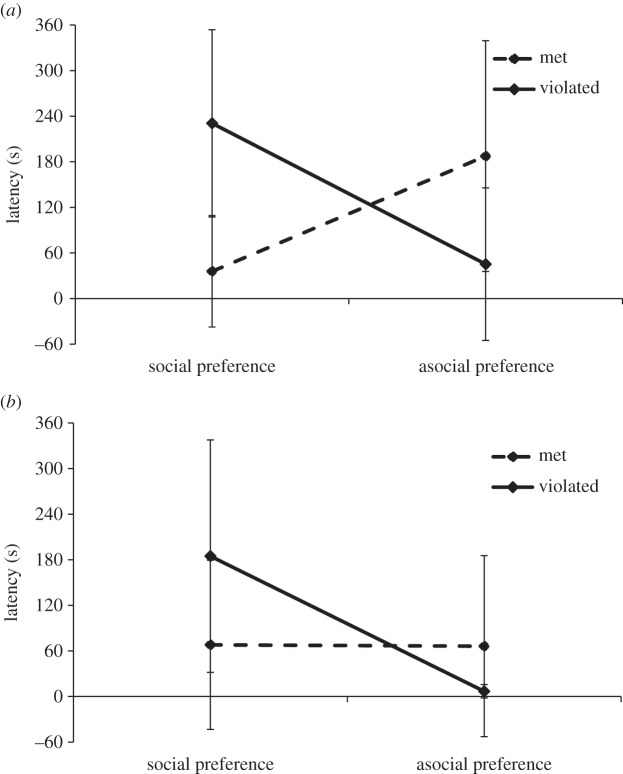
Latency according to learning style preference and congruence: (*a*) 3-year-olds and (*b*) 5-year-olds.

Tukey HSD follow-up tests were conducted between children who received their requested learning style versus being allocated to their non-preferred learning style, for both learning style choices (social versus asocial), for each age group. Three-year-olds in the chose-social–received-social condition (*M* = 35 s; s.d. = 73 s) were significantly faster at the task than those in the chose-social–received-asocial condition (*M* = 231 s; s.d. = 123 s), *p* < 0.001. Also, 3-year-olds in the chose-asocial–received-social condition (*M* = 45 s; s.d. = 100 s) were significantly faster than those in the chose-asocial–received-asocial condition (*M* = 188 s; s.d. = 152), *p* = 0.014. Similarly, 5-year-olds in the chose-social–received-social condition (*M* = 68 s; s.d. = 111) were significantly faster than those in the chose-social–received-asocial condition (*M* = 185; s.d. = 153), *p* = 0.011. However, interestingly, there was no significant difference between 5-year-olds in the chose-asocial–received-social condition (*M* = 7 s; s.d. = 9 s) and the chose-asocial–received-asocial condition (*M* = 66 s; s.d. = 119 s), *p* = 0.781. The large standard deviation scores for latencies reflect that 28% of all children were unsuccessful and had their score capped at 360 s. To account for this and the fact of uneven cell-sizes and variances, it was especially important to take the robust measures mentioned above; levels of lack of success are reported with the further statistical description of conditions in the electronic supplementary material, S1. Taken together these results indicate that for 3-year-olds, those who received social input, whether choosing it or not, were faster at the task than children who learned asocially. This effect held for 5-year-olds who chose to learn socially, but 5-year-olds who chose to learn asocially were equally fast at the task no matter the learning style they were allocated to receive (as depicted in figure 2).

#### (ii) Adults' task latency

An ANOVA of AF difficulty, stated learning preference, and having the preference met or violated was used to examine main effects, where *n* was large enough to compare, and as with children the first trial data were used, *F*_3,48_ = 8.92, *p* *<* 0.001. Non-parametric statistics were employed to help further exposition about how learning choice and learning congruence affected task performance, for each AF (as with children, equivalent robust tests were run, see the electronic supplementary material, S3).

Adults completed the easy SB significantly faster than the hard PP, *F*_1,48_ = 18.19, *p* < 0.001. There was a significant difference in task latency between adults who had their preference met (*M* = 6 s, s.d. = 9 s) and those who were allocated to receive their non-preferred learning style (*M* = 15 s, s.d. = 18 s), *F*_1,48_ = 7.55, *p* = 0.008. There was also no difference in latency between those who requested social (*M* = 11 s, s.d. = 14 s) versus asocial learning (*M* = 8 s, s.d. = 13 s), *F*_1,48_ = 0.38, *p* = 0.548.

Kruskal–Wallis tests were employed to compare each of the preferred learning strategy congruency conditions for each AF (descriptive statistics presented in table 2). For the easy SB, no significant difference was found in task latency *χ*^2^(3, *N* = 26) = 5.52, *p* = 0.137, likely resulting from a floor-effect as all adults were quick at completing this task. For the hard PP task, a significant difference was found in latency, *χ^2^*(3, *N* = 26) = 13.14, *p* = 0.004. Like children, adults appeared faster when they received a social demonstration, with those choosing to learn asocially being the fastest after a social demonstration. The chose-asocial–received-asocial condition contained a single individual, so no comment could be made about this condition.

**Table 2.** Adult task latency by chosen learning preference and learning style received.

	*n*	mean rank	mean	s.d.	min.	max.
easy AF, SB						
chose-social–received-social	7	12.07	2.57	1.13	2.00	5
chose-social–received-asocial	9	17.67	4.11	2.57	2.00	10
chose-asocial–received-social	6	10.67	2.25	0.50	2.00	3
chose-asocial–received-asocial	4	10.88	2.33	0.82	2.00	4
hard AF, PP						
chose-social–received-social	12	9.04	8.08	4.6	2.00	18
chose-social–received-asocial	7	21.64	34.42	17.09	15.00	20
chose-asocial–received-social	6	11.83	15.5	18.57	5.00	53
chose-asocial–received-asocial	1	20	26.00			

#### (iii) Adults' performance on the transformer task

*Task latency.* A multiple regression was used to examine the effects of learning preference, preference congruence, the interaction of these effects, as well as the assessment of task difficulty on latency (table 3). The model was found to be significant, *R*^2^ = 0.55, 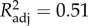, *F*_4,47_ = 14.32, *p* < 0.001. Assessment of difficulty was found to be significantly positively correlated with latency; those who assessed the task as more difficult took longer to complete it. It was also found that those choosing social learning were significantly faster in completing the task than those choosing asocial learning.

**Table 3.** Multiple regression on the predictors of adults' latency (*N* = 52).

variable	mean	s.e.	sr^2^	*β*	*t*	*p*-value
assessment of difficulty			0.49	0.86	7.12	0.001**
learning preference			0.05	−0.27	−2.21	0.032*
social	5.72	0.24				
asocial	6.73	0.35				
preference congruence			0.02	−0.13	−1.25	0.216
met	5.99	0.28				
violated	6.46	0.26				
learning pref. × preference cong.			0.01	−0.11	1.02	0.314
chose-social–received-social	5.68	0.48				
chose-social–received-asocial	5.76	0.45				
chose-asocial–received-social	7.16	0.35				
chose-asocial–received-asocial	6.30	0.31				

*p < 0.05, **p < 0.001.

## 4. Discussion

Three main findings arise from this study. First, 3-year-olds, 5-year-olds and adults all appear to have a preference for social learning when presented with a free choice in learning style to solve a novel puzzle box problem. Second, we see a developmental shift between 3 and 5 years in the knowledge of which type of information one needs to perform well at a task. Third, adults were accurate in perceiving how difficult a task would be, and those perceiving it as more difficult asked to learn socially.

All age groups, 3-year-olds, 5-year-olds and adults, showed a preference to learn socially rather than asocially when presented with a novel puzzle box. Such a finding has implications for our understanding of innovation as following the witnessing of social information the overwhelming response is for individuals to repeat the witnessed behaviour [3,14], ultimately resulting in less innovation. Thus, our design shows that a preference for social learning over asocial learning can be added to the list of factors impeding innovation. Furthermore, we show experimentally that individuals seek such information when faced with a new problem. It has only been by providing our participants with a choice about how they attempt the tasks that we have been able to identify this preference, and the resulting effect these preferences have on performance. Interestingly, the results of our adult sample contrast with the findings of Mesoudi [24] who found that over three quarters of a sample of adults were more likely to use asocial learning than social learning in a complex virtual artefact-design task [24]. Adults in this study did move from social learning to more asocial learning between trials 1 and 2, but not to the scale seen in Mesoudi [24]. The differences in findings could be owing to a number of factors including the fact that the tasks in this study were not virtual tasks as in Mesoudi's arrow head design task, and in Mesoudi [24] adult participants had 90 trials, whereas in this study participants had one trial on each task.

The influence of task difficulty and trial on the preference for social learning was complicated, varying between children and adults. Children's preferences were not influenced by described task difficulty in either trial. By contrast, on their second trial, adults demonstrated no dominant preference for social learning, as social learning was requested 50% of the time. During this second trial adults' preferences were modulated by task difficulty, with a marginal effect suggesting adults were more likely to request a demonstration when presented with the ‘hard’ AF compared with the ‘easy’ AF. This influence of task difficulty was repeated in the adults' learning style choice when presented with the Transformer task, as adults who chose social learning rated the task as more difficult than adults who chose asocial learning. Thus, social learning preference becomes selective, as adults make a contextual choice about how to learn based on their perception about the difficulty of the task. We can hypothesize from these findings that we are more likely to see innovation on tasks that individuals believe are easy, or feel expert in; such ideas are ripe for further exploration.

A second critical finding related to how the preference and use of these different learning strategies affected performance on the tasks. The 5-year-olds in this study showed a level of sensitivity to the tasks presented to them. By 5 years of age, children did not simply ask for social learning, but showed more strategic learning choices in general. Three-year-olds' performance was dictated by the type of learning they received: irrespective of what they asked for, those receiving social learning showed better performance than those who learned asocially. However, while this trend held for 5-year-olds who requested social learning, those 5-year-olds who asked to learn asocially were faster at the task no matter which learning style they were allocated, social or asocial. By 5 years, children are selective in choosing a learning strategy that is effective for them, a propensity not seen at 3 years. This result may be regarded more tentatively as it relies on a smaller sample owing to the more dominant preference for social learning in 5-year-olds, a consequence of an informative design in which the relationship between preference and performance could be studied together. Future research can now profitably implement a non-quasi experimental design to avoid this limitation to replicate this effect. Adults' performance on the Transformer task further exemplifies this shift towards greater knowledge of what information is necessary to perform well. Adults correctly assessed the difficulty of the task in terms of their performance, requesting social information when they assessed it as more difficult and asocial learning when they assessed it as less difficult. Such a finding complements previous research, which has highlighted that innovation is a relatively late-developing capacity [18–20] and modification is a rare response for children [16]. Thus, where it had previously been assumed children would become better social information users with age [28], in fact they become better information users in general with age, an idea in line with recent arguments that social learning is an extension of more general learning processes [29]. It is unclear if the 5-year-olds who are requesting asocial learning could see the solution or had learned that they were good at attempting tasks themselves; again this is an avenue for future research.

In summary, cultural evolution requires the interplay of sustaining traditions through social learning and creating new behaviours through innovation [1]. The current results show that, in an open choice design, young children and adults have a preference for social learning rather than asocial learning. Thus, our reliance on social learning is supported by a dominant preference to receive it. Importantly, for cultural evolution, we also need individuals to create alternatives, and thus to prefer asocial learning. By 5 years, we see a small but critical group of asocial learners, whose skills at asocial learning are as effective as social learners. This may be the first evidence in the literature of a more general understanding of information requirements and use, which develops with age. Pertinent questions for our future research relate to what degree asocial learning is a trait, stable across domains, or if it varies substantially in different contexts; and whether this preference is associated with particular personality styles.

## Supplementary Material

Supplementary material

## References

[1] Legare C, Nielsen M 2015 Imitation and innovation: the dual engines of cultural learning. Trends Cognit. Sci. 19, 688–699. (10.1016/j.tics.2015.08.005) 26440121

[2] Dean L, Vale G, Laland K, Flynn E, Kendal R 2014 Human cumulative culture: a comparative perspective. Biol. Rev. 89, 284–301. (10.1111/brv.12053) 24033987

[3] Carr K, Kendal RL, Flynn E in press. Eureka!: What is innovation, how does it develop and who does it? Child Dev. 10.1111/cdev.12549 PMC5053256 27241583

[4] Mesoudi A et al. 2013 The cultural evolution of technology and science. In Cultural evolution: society, technology, language, and religion (eds Richerson P, Christiansen M), pp. 192–216. Cambridge, MA: MIT Press.

[5] Reader SM, Morand-Ferron J, Flynn E 2016 Animal and human innovation: novel problems and novel solutions. Phil. Trans. R. Soc. B 371, 20150182 (10.1098/rstb.2015.0182) 26926273 PMC4780525

[6] Beck SR, Williams C, Cutting N, Apperly IA, Chappell J 2016 Individual differences in children's innovative problem-solving are not predicted by divergent thinking or executive functions. Phil. Trans. R. Soc. B 371, 20150190 (10.1098/rstb.2015.0190) 26926280 PMC4780532

[7] Reader SM, Laland KN 2003 Animal innovation: an introduction. In Animal innovation (eds Reader SM, Laland KN), pp. 3–35. Oxford, UK: Oxford University Press.

[8] Meltzoff A, Moore M 1983 Newborn infants imitate adult facial gestures. Child Dev. 54, 702–709. (10.2307/1130058) 6851717

[9] Carr K, Kendal RL, Flynn E 2015 Imitate or innovate? Children's innovation is influenced by the efficacy of observed behaviour. Cognition 142, 322–332. (10.1016/j.cognition.2015.05.005) 26072278

[10] Williamson RA, Meltzoff AN, Markman EM 2008 Prior experiences and perceived efficacy influence 3-year-olds’ imitation. Dev. Psychol. 44, 275–285. (10.1037/0012-1649.44.1.275) 18194026 PMC2259446

[11] Gardiner AK, Bjorklund DF, Greif ML, Gray SK 2012 Choosing and using tools: prior experience and task difficulty influence preschoolers’ tool-use strategies. Cognit. Dev. 27, 240–254. (10.1016/j.cogdev.2012.05.001)

[12] Pinkham AM, Jaswal VK 2011 Watch and learn? Infants privilege efficiency over pedagogy during imitative learning. Infancy 16, 535–544. (10.1111/j.1532-7078.2010.00059.x) 32693552

[13] Williamson RA, Meltzoff AN 2011 Own and others’ prior experiences influence children's imitation of causal acts. Cognit. Dev. 26, 260–268. (10.1016/j.cogdev.2011.04.002) 21966091 PMC3181112

[14] Wood LA, Kendal RL, Flynn EG 2013 Copy me or copy you? The effect of prior experience on social learning. Cognition 127, 203–213. (10.1016/j.cognition.2013.01.002) 23454793

[15] Wood LA, Kendal RL, Flynn EG 2013 Whom do children copy? Model-based biases in social learning. Dev. Rev. 33, 341–356. (10.1016/j.dr.2013.08.002)

[16] Whiten A, Flynn E 2010 The transmission and evolution of experimental microcultures in groups of young children. Dev. Psychol. 46, 1694–1709. (10.1037/a0020786) 20822212

[17] Flynn E, Whiten A 2008 Cultural transmission of tool use in young children: a diffusion chain study. Social Dev. 17, 699–718. (10.1111/j.1467-9507.2007.00453.x)

[18] Beck SR, Apperly IA, Chappell J, Guthrie C, Cutting N 2011 Making tools isn't child's play. Cognition 119, 301–306. (10.1016/j.cognition.2011.01.003) 21315325

[19] Hanus D, Mendes N, Tennie C, Call J 2011 Comparing the performances of apes (*Gorilla gorilla, Pan troglodytes, Pongo pygmaeus*) and human children (*Homo sapiens*) in the floating peanut task. PLoS ONE 6, e19555 (10.1371/journal.pone.0019555) 21687710 PMC3110613

[20] Nielsen M 2013 Young children's imitative and innovative behavior on the floating object task. Infant Child Dev. 22, 44–52. (10.1002/icd.1765)

[21] German TP, Defeyter MA 2000 Immunity to functional fixedness in young children. Psychon. Bull. Rev. 7, 707–712. (10.3758/BF03213010) 11206213

[22] Bonawitz E, Shafto P, Gweon H, Goodman ND, Spelke E, Schulz L 2011 The double-edged sword of pedagogy: instruction limits spontaneous exploration and discovery. Cognition 120, 322–330. (10.1016/j.cognition.2010.10.001) 21216395 PMC3369499

[23] Cutting N, Apperly IA, Chappell J, Beck SR 2014 The puzzling difficulty of tool innovation: why can't children piece their knowledge together? J. Exp. Child Psychol. 125, 110–117. (10.1016/j.jecp.2013.11.010) 24530037

[24] Mesoudi A 2011 An experimental comparison of human social learning strategies: payoff-biased social learning is adaptive but underused. Evol. Hum. Behav. 32, 334–342. (10.1016/j.evolhumbehav.2010.12.001)

[25] Hopper LM, Lambeth SP, Schapiro SJ, Whiten A 2008 Observational learning in chimpanzees and children studied through ‘ghost’ conditions. Proc. R. Soc. B 275, 835–840. (10.1098/rspb.2007.1542) PMC2596905 18182368

[26] Flynn E, Whiten A 2012 Experimental 'microcultures’ in young children: identifying biographic, cognitive, and social predictors of information transmission. Child Dev. 83, 911–925. (10.1111/j.1467-8624.2012.01747.x) 22417384

[27] Hopper LM, Flynn EG, Wood LA, Whiten A 2010 Observational learning of tool use in children: investigating cultural spread through diffusion chains and learning mechanisms through ghost displays. J. Exp. Child Psychol. 106, 82–97. (10.1016/j.jecp.2009.12.001) 20064644

[28] McGuigan N, Makinson J, Whiten A 2011 From over-imitation to super-copying: adults imitate irrelevant aspects of tool use with higher fidelity than young children. Br. J. Psychol. 102, 1–18. (10.1348/000712610X493115) 21241282

[29] Heyes C 2012 What's social about social learning? J. Comp. Psychol. 136, 195–202. 10.1037/a0025180 21895355

