# Supplementary material for: Selectivity in social and asocial learning: investigating the prevalence, effect and development of young children's learning preferences
Source: Philos Trans R Soc Lond B Biol Sci. 2016 Mar 19;371(1690):20150189. doi: 10.1098/rstb.2015.0189 (PMC4780531; doi:10.1098/rstb.2015.0189)
Supplement: Supplementary material [file rstb20150189supp1.pdf]

2016 *Phil. Trans. R. Soc B* **371** doi: 10.1098/rstb.2015.0189

Selectivity in social and asocial learning: Investigating the prevalence, effect and development of young children's learning preferences

Emma Flynn, Cameron Turner and Luc-Alain Giraldeau

Supplementary Materials

Contents

1. Description of latency by condition for children (first trial data)
2. Analysis which shows that Artificial Fruit (AF) difficulty does not significantly interact with other factors for children's latency scores
    a. Full factorial untransformed
    b. Full factorial transformed
    c. Age group * AF difficulty untransformed
    d. Age group * AF difficulty transformed
3. Alternative analyses to ensure robustness

    Children's task latency tests

    a. Children's Levene's test
    b. Bootstrapping and Games-Howell adjusted follow-up test
    c. Nonparametric equivalent follow-up tests
    d. Analysis with log transformation

    Adult task latency tests

    e. Adult's Levene's test
    f. Analysis with log transformation

2. Description of latency by condition for children (first trial data)

	n	Skew	Log. Skew	Kurt.	Log. Kurt.	Med.	Min.	Max.	Mean	SD	% Unsucc.
Three-year-olds	72	.415	-.40	-1.70	-1.43	68	2	360	153	158	33%
Chose-social-received-social	24	2.65	.65	6.52	-.94	6	2	360	52	101	13%
Hard AF	12	1.80	-.14	1.74	-.39	39	2	360	94	129	25%
Easy AF	12	3.45	2.90	11.95	9.05	2	2	93	10	26	0%
Chose-social-received-asocial	24	-1.43	-2.09	.54	3.39	360	12	360	285	124	68%
Hard AF	12	-1.22	-1.88	.20	2.46	360	26	360	272	126	58%
Easy AF	12	-1.92	-2.46	2.32	5.65	360	12	360	298	126	75%
Chose-asocial-received-social	12	2.97	.70	9.24	-.83	7	2	355	49	101	0%
Hard AF	6	2.06	-.20	4.35	-.13	34	2	355	90	135	0%
Easy AF	6	2.45	2.45	6.00	6	2	2	33	7	13	0%
Chose-asocial-received-asocial	12	.02	-.95	-2.01	-.18	7	2	360	197	153	42%
Hard AF	6	-.34	-.78	-2.57	-1.75	268	33	360	223	158	50%
Easy AF	6	.40	-.83	-2.01	-.89	140	7	360	171	158	33%

Five-year-olds	66	.94	.03	-.85	-1.57	21	2	360	113	143	21%
Chose-social-received-social	24	1.77	.48	2.13	-1.39	7	2	360	70	112	8%
Hard AF	12	1.20	-.24	.266	-1.28	6	2	360	118	129	17%
Easy AF	12	3.46	2.99	11.99	9.58	3	2	244	23	70	0%
Chose-social-received-asocial	24	-.14	-1.11	-1.89	.14	78	2	360	203	154	46%
Hard AF	12	-.03	-.78	-2.02	-.42	206	22	360	211	142	42%
Easy AF	12	-.16	-.62	-2.24	-1.43	245	2	360	195	171	50%
Chose-asocial-received-social	6	1.87	.74	3.53	-.62	5	2	25	8	9	0%
Hard AF	3	1.01	-.40	.	.	10	3	25	13	11	0%
Easy AF	3	1.73	1.73	.	.	2	2	6	3	2	0%
Chose-asocial-received-asocial	12	2.01	.51	3.03	-.96	15	2	360	68	119	8%
Hard AF	6	.97	.07	-1.09	-2.49	49	4	360	123	154	17%
Easy AF	6	1.48	.27	2.13	-2.45	10	2	44	14	16	0%

3. Artificial Fruit (AF) difficulty does not significantly interact with other factors for children's latency scores (first trial).

   a. *Full factorial untransformed*

	df	*F*	*p*
Model	15	6.24	.001**
Age group	1	6.37	.013*
AF difficulty	1	5.18	.025*
Preference congruence	1	2.90	.019*
Learning preference	1	9.77	.002*
Age group * learning preference	1	1.31	.254
Age group * preference congruence	1	.02	.897
Age group * AF difficulty	1	.04	.843
Learning preference * preference congruence	1	38.67	.001**
Learning preference * AF difficulty	1	.21	.650
Preference congruence * AF difficulty	1	1.92	.169
Age group * learning preference * preference congruence	1	4.16	.044*
Age group * preference congruence * AF difficulty	1	.29	.593
Age group * learning preference * AF difficulty	1	.14	.705
Learning preference * preference congruence * AF difficulty	1	.43	.515
Age group * learning preference * preference congruence * AF difficulty	1	.77	.382

$N = 138$, $R^2 = .434$, $**p < .001$, $* p <.05$

b. Full factorial transformed

	df	*F*	*p*
Model	15	11.50	.001**
Age group	1	8.76	.004*
AF difficulty	1	29.33	.001**
Preference congruence	1	1.58	.212†
Learning preference	1	12.68	.001**
Age group * learning preference	1	4.14	.044*†
Age group * preference congruence	1	.06	.062
Age group * AF difficulty	1	.27	.271
Learning preference * preference congruence	1	64.38	.001**
Learning preference * AF difficulty	1	.01	.960
Preference congruence * AF difficulty	1	2.16	.144
Age group * learning preference * preference congruence	1	5.18	.025*
Age group * learning preference * AF difficulty	1	.54	.463
Learning preference * preference congruence * AF difficulty	1	6.48	.012*†
Age group * learning preference * preference congruence * AF difficulty	1	1.72	.193

$N = 138$, $R^2 = .535$, $^{**}p < .001$, $^{*}p < .05$, †Different in significance from untransformed

*c. Age group * AF difficulty untransformed*

	df	*F*	*p*
Model *non-significant*	3	2.22	.089
Age group	1	2.55	.113*
AF difficulty	1	4.028	.046*
Preference congruence	1	.14	.706

N = 138, R^2 = .047, ***p* < .001, * *p* <.05

*d. Age group * AF difficulty transformed*

	df	*F*	*p*
Model	15	7.51	.001**
Age group	1	2.04	.155*
AF difficulty	1	20.28	.001**
Preference congruence	1	.42	.521†

N = 138, R^2 = .144, ***p* < .001, * *p* <.05, †Different in significance from untransformed

4. Alternative analyses to ensure robustness

a. Levene's test of the children's data

ANOVA of children's task latency was found to violate the assumption of equal variances by Levene's test, $F(15, 122) = 3.93$, $p < .001$, first trial. Below are measures taken to insure the robustness of findings.

b. *Bootstrapping and Games-Howell adjusted follow-up test*

	Mean difference	*p*	Bias	BCa 95% Confidence Interval Lower	Upper
Three-year-olds					
Chose-social-received-asocial Chose-social-received-social	233.04	.001**	-.72	165.02	291.72
Chose-asocial-received-asocial Chose-asocial-received-social	148.41	.051*[a]	1.31	36.10	255.41
Five-year-olds					
Chose-social-received-asocial Chose-social-received-social	132.83	.008*	-.94	56.58	205.24
Chose-asocial-received-asocial Chose-asocial-received-social	60.25	.347	1.03	4.24	133.19

$^{**}p < .001$, $^{*}p < .05$, a. Marginal significance

c. Nonparametric equivalent follow-up tests

	Mean rank	Mann-Whitney U	Wilcoxon W	*p*
Three-year-olds				
Chose-social-received-asocial	34.21	55.00	355.00	.001**
Chose-social-received-social	14.79			
Chose-asocial-received-asocial	8.13	19.50	97.50	.001**
Chose-asocial-received-social	16.88			
Five-year-olds				
Chose-social-received-asocial	31.31	124.50	424.00	.001**
Chose-social-received-social	17.69			
Chose-asocial-received-asocial	10.50	24.00	45.00	.291
Chose-asocial-received-social	7.50			

d. Analyses with Log transformation

Log transformation was found to successfully correct for violation of equality of error variance, in the children's data, $F(7, 64) = 1.10$, $p = .376$, first trial.

Children: analysis of variance on task latency

	df	*F*	*p*
Model	8	17.87	.001**
Age group	1	8.09	.005*
AF difficulty	1	34.87	.001**
Preference congruence	1	1.46	.230†
Learning preference	1	2.94	.001**†
Age group * learning preference	1	1.33	.053
Age group * preference congruence	1	.02	.812
Learning preference * preference congruence	1	39.21	.001**
Age group * learning preference * preference congruence	1	4.22	.030*

$N = 137$, $R^2 = .526$, $^{**}p < .001$, $^{*}p < .05$, †Different in significance from untransformed

Follow-up test

	Mean difference	Std. Error	*p*	95% Confidence Interval Lower	Upper
Three-year-olds					
Chose-social-received-asocial Chose-social-received-social	1.29	.19	.001**	.92	1.68
Chose-asocial-received-asocial Chose-asocial-received-social	1.06	.27	.001**	.53	1.59
Five-year-olds					
Chose-social-received-asocial Chose-social-received-social	.83	.27	.003*	.28	1.34
Chose-asocial-received-asocial Chose-asocial-received-social	.49	.37	.200	-.26	1.24

$^{**}p < .001$, $^{*}p < .05$

e. Levene's test with adult's data

ANOVA of adults' task latency was found to violate the assumption of equal variances by Levene's test, $F(7, 122) = 3.54$, $p = .004$, first trial, Artificial Fruit task. Below are measures taken to insure the robustness of findings; as follow-up tests were already non-parametric and exploratory, only a log transformed model is presented

f. Analyses with log transformation

Log transformation was found to successfully correct for violation of equality of error variance, in the adult data, $F(7, 44) = 1.06$, $p = .405$, first trial Artificial Fruit task.

Adults: analysis of variance on task latency

	df	*F*	*p*
Model	4	27.67	.001**
AF difficulty	1	91.16	.001**
Preference congruence	1	5.23	.027*†
Learning preference	1	.93	.340
Learning style received	1	9.99	.003*

$N = 137$, $R^2 = .526$, ** $p < .001$, * $p < .05$, †Different in significance from untransformed
